# Genetic diversity and population structure analysis in a large collection of *Vicia amoena* in China with newly developed SSR markers

**DOI:** 10.1186/s12870-021-03330-w

**Published:** 2021-11-20

**Authors:** Feifei Wu, Shangxiong Zhang, Qiu Gao, Fang Liu, Jianli Wang, Xianguo Wang

**Affiliations:** 1 College of Grassland Science and Technology, China Agricultural University, Beijing, 100193 China; 2 National Herbage Germplasm Conservation center of China, Beijing, 10025 China; 3 Grass and Science Institute, Heilongjiang Academy of Agricultural Science, Harbin, 150086 China

**Keywords:** *Vicia amoena*, SSR development, genetic variation, population structure

## Abstract

*Vicia amoena* is a high-nutritional quality forage similar to alfalfa. However, studies on the genetic background of *V. amoena* are scarce. In the present study, the genetic variation of 24 *V. amoena* populations was assessed with newly developed simple sequence repeat (SSR) markers. A total of 8799 SSRs were identified in the *V. amoena* genomic-enriched sequences, and the most abundant repeat number was four. A total of 569 sampled individuals were assayed to evaluate the genetic diversity of the *V. amoena* populations based on 21 polymorphic SSR primers. The polymorphism information content (PIC) ranged from 0.896 to 0.968, with an average of 0.931, which indicated that the markers were highly informative. Based on analysis of molecular variance, 88% of the variance occurred within populations, and the remaining 12% of the variance occurred among populations. The high degree of gene flow (Nm= 4.958) also showed slight differentiation among the *V. amoena* populations. The *V. amoena* populations were mainly clustered by steppe and mountain habitats based on principal coordinate analysis (PCoA) and STRUCTURE analysis. This indicated that the elevation and special habitat of geographical origins may be important factors affecting the clustered pattern of *V. amoena* populations. Neighbour-joining (NJ) analysis did not separate the populations well by geographical origin, which indicated that the genetic structure of *V. amoena* was complex and needs further study. Overall, our results showed that the newly developed SSR markers could benefit the *V. amoena* research community by providing genetic background information to help establish a foundation for breeding improvement and germplasm resource conservation.

**Supplementary Information:**

The online version contains supplementary material available at 10.1186/s12870-021-03330-w.

## Background

*Vicia amoena* is an herbaceous, allotetraploid (2n=24), perennial legume species native to Eastern Asia (Siberia, Mongolia, China, Japan, and Korea) that is especially widely dispersed in northern China [1, 2]. It has high nutritional quality, strong abiotic stress tolerance, and wide adaptability. The protein content and the amino acid content of *V. amoena* are comparable to those of alfalfa (*Medicago sativa*) [3]. Moreover, *V. amoena* is also used as a traditional Chinese medicinal herb to treat oedema, rheumatoid arthritis and contracture [4]. However, genetic research on this important forage legume is scarce, with most researchers instead focusing on its chemical components. Unravelling the genetic diversity and population structure of *V. amoena* is very important for understanding its genetic background, which is a prerequisite for future genetic research, breeding programme development and genetic resource conservation.

Microsatellites or simple sequence repeat (SSR) markers are a powerful molecular method for quantifying genetic variation in plants due to their high polymorphism [5]. SSR markers are characterized by repeated sequences comprising mono-, di-, tri-, tetra-, penta- or hexa-nucleotide units that are characterized by tandem repeats (1-10 nucleotide motifs) that exhibit locus-specific codominance and high heterozygosity, are distributed throughout the genome, and are easier to detect than other molecular markers [6]. Microsatellite markers have been successfully used in the assessment of many plants, e.g., *Vicia faba* [7, 8], *Campomanesia adamantium* [9], *Populus deltoides* [10], *Olea europaea* [11], and *Cunninghamia lanceolata* [12].

Overall, SSRs are one of the most informative molecular markers for plant genetic research, but the isolation of SSR markers traditionally based on probe hybridization is an experimentally demanding, labour-intensive, and economically costly process [13]. Advancements in sequencing and bioinformatic analysis techniques have provided good opportunities for generating new SSR markers. For example, next-generation sequencing (NGS) technology is a powerful tool that can be used for fast and cost-effective SSR discovery [14, 15]. To date, a large number of SSR markers have been developed by high-throughput sequencing in many plants, such as *Medicago sativa* [16], *Vicia sativa* [15], *Elymus sibiricus* [17], *Onobrychis viciifolia* [18], *Angelica gigas* [19], *Lentinula edodes* [20], and *Spondias tuberosa* [21].

In the present study, we developed SSR markers using the HiSeq 4000 PE150 sequencing platform. We then used 21 polymorphic pairs to analyse the genetic diversity and population structure of 24 *V. amoena* populations (569 total individuals) in China, which may support studies on molecular diversity and breeding programmes. Our goals are (1) to assess the validity of these newly developed SSR markers and (2) to obtain an accurate representation of the genetic diversity and population structure of *V. amoena*.

## Material and methods

### Plant materials and DNA isolation

A total of 569 individuals from 24 sites throughout the natural distribution of *V. amoena* in China were collected in the present study (Table 1). Of these individuals, 281 individuals from 13 populations were collected in the field. The other 288 individuals from 11 sites were obtained from seeds provided by the National Herbage Germplasm Conservation Centre of China (Beijing, China). Genomic DNA was extracted from fresh or silica gel-dried leaf tissues using a Plant Genomic DNA Extraction Kit (Tiangen, Beijing, China) according to the manufacturer’s protocol.

**Table 1:** The detail information of *Vicia amoena* populations in this study

Population ID	Origin	Source	Latitude(N)	Longitude(E)	Altitude(m)	Sample size
N50	Oroqen Banner, Inner Mongolia	Field collection	50°16'28"	124°15'17"	369.23	32
STG	Songtagou, Inner Mongolia	Field collection	48°8'14"	123°21'47"	304.43	18
YDZ	Yadong town, Inner Mongolia	Field collection	48°28'5"	123°48'6"	294.07	11
HEB	Harbin, Heilongjiang	Field collection	45°53'51"	126°27'8"	159.6	27
XLT	Xeltala, Inner Mongolia	Field collection	49°20'23"	119°59'43"	624.97	27
QHA	Minhe, Qinghai	Field collection	36°11'39"	102°44'21"	2044.57	14
QHB	Minhe, Qinghai	Field collection	36°5'	102°44'27"	2301.37	12
SJ	Yuncheng, Shanxi	Field collection	35°8'21"	111°26'22"	1040.21	20
YX	Taiyuan, Shanxi	Field collection	38°11'51"	112°49'54"	1221.43	24
YHT	Yihuta, Inner Mongolia	Field collection	43°4'55"	122°15'52"	241.83	25
ZQ	Zhalute, Mongolia	Field collection	44°56'42"	120°21'23"	673.81	28
MQ	Morin Dawa, Inner Mongolia	Field collection	48°41'54"	124°32'8"	254.94	17
ZD	Zhaodong, Heilongjiang	Field collection	46°14'30"	125°27'58"	187.32	26
M99	Chifeng, Inner Mongolia	National Herbage Germplasm Conservation Centre of China	43°07′41″	119°03′17″	-	30
NM03	Liangcheng, Inner Mongolia	National Herbage Germplasm Conservation Centre of China	40°34'59″	112°19'55″	1590	15
ZX476	Wutai Mountain, Shanxi	National Herbage Germplasm Conservation Centre of China	38°52'43″	113°39'37″	-	30
ZX541	Ling Mountain, Beijing	National Herbage Germplasm Conservation Centre of China	39°59'10"	115°29'8"	-	30
ZX562	Yizhou, Shanxi	National Herbage Germplasm Conservation Centre of China	38°56'6"	112°26'30"	-	24
B514	Ershan, Inner Mongolia	National Herbage Germplasm Conservation Centre of China	47°17'19″	119°49'2"	-	30
B515	Hulunbeier, Inner Mongolia	National Herbage Germplasm Conservation Centre of China	48°14′	120°0′28″	-	28
B516	Hulunbeier, Inner Mongolia	National Herbage Germplasm Conservation Centre of China	48°43'41″	118°47'38″	-	30
ZX986	Beijing	National Herbage Germplasm Conservation Centre of China	40°31′13″	115°46′04″	998.4	26
ZX987	Zhangjiakou, Hebei	National Herbage Germplasm Conservation Centre of China	40°34′39″	115°47′	1362	15
ZX1141	Beijing	National Herbage Germplasm Conservation Centre of China	40°10′18″	116°13′24″	49	30

### SSR marker detection, identification, and primer design

An Illumina paired-end library was constructed by the NEBNext® Ultra™ II DNA Library Preparation Kit (New England Biolabs (Beijing) Ltd., China) and sequenced on the Illumina HiSeq 4000 PE150 sequencing platform. Approximately 17.5 Gb of raw data was generated, and the raw sequence reads were filtered for primer/adaptor sequences and low-quality reads with the NGS QC Tool Kit [22]. Sequencing reads were assembled using SPAdes 3.6.1 software [23] with the parameter Kmer=95, and 198,659 contigs were finally obtained.

MISA software [24, 25] was used to identify unique reads containing microsatellite repeats. The search was performed for a minimum repeat number of 5, 4, 3, 3 and 3 for di-, tri-, tetra-, penta-, and hexa-nucleotides, respectively. Primers were designed on the basis of flanking sequences of SSR microsatellite loci by using Primer 3. The parameters of primer design were set as follows: the primer size was between 18 and 25 bp with an optimal size of 22 bp, the annealing temperature was between 55 and 65 °C with the optimal temperature of 60 °C, the PCR product size was between 80 and 300 bp, and default values were selected for other settings.

### M13-SSR PCR amplification

Twenty-one SSRs were selected through a preliminary experiment, and this number of markers was suitable for evaluating plant genetic diversity [11, 26, 27]. Twenty-one primer pairs (Table 2) that successfully amplified fragments in the 569 individuals were further characterized for polymorphisms using the M13-SSR PCR protocol. There were three primers in the M13-SSR PCR system: a forward primer, a reverse primer with an M13-tail (5’-CACGACGTTGTAAAACGAC-3’) at the 5’ end, and a fluorescently labelled M13 universal primer. The first two primers were synthesized by Sangon Biotech (Shanghai, China) Co., Ltd., and the third primer was synthesized by Thermo Fisher Scientific (Shanghai, China). The four fluorescently labelled primers were FAM, NED, VIC, and ROX.

**Table 2:** The detail information of SSR primers used in the population genetic study

Primers ID	motifs	Forward primers (5' to 3’)	Reverse primers (5' to 3’)	Target fragment length (bp)	Annealing temperature (°C)
VA 1	(TTG)8	GTTTGGGAAAGAAACGTCGTCA	ACCGAACACTTAGTGTGCAAGT	143-156	56
VA 4	(TTA)8	ACCGTACAATGTAAGGGTAAACGA	TCCACTTAGTCAATTAGCCACACA	189-243	55
VA 8	(TGT)7	GAAACCCAATGTTCTTGCGGAA	AATACCCTTGCCTTACGCGTAA	264-273	56
VA 9	(TG)9	CTTATGTAGCTGGCGTGTTTGT	TTGACCTTGGATTTGGGCCATA	192-224	56
VA 11	(TG)7	CTGATCTAATAACTTGGCGCGC	TGTCGGTCTGTTTGAGTGAACT	261-281	56
VA 14	(TCT)7	CAACACGGCGAAAACGATGATA	TACGCATCACACAAACCACAAC	232-247	56
VA 16	(TCA)7	CTGTACCCGAGGCTCTGAGT	AAGACAAGCAAGAAGTTGTCGC	166-187	56
VA 19	(TC)7	CTGACCCAAGTAATCCTCTCCG	TGGACGGTGATGTTTTGGATCT	127-145	56
VA 21	(TAA)7	GGAGCTAAAGCCACTCGTGTAA	CCATACGCCCCCACATTTTTAC	242-257	56
VA 23	(TA)8	TTTGGTTTGGTCCCCTTGTACA	TTCTGACCCACTTCAGGTTCTG	182-196	56
VA 25	(TA)10	TTCTGACTCCGATTTTGCTGGA	CAAGAGTTTGCTTGCCACTGC	113-145	58
VA 26	(GTTA)7	TAGAAGAGAAGCAGACAAGGGC	ACAACAAATTCTAGTACAGGTCTCA	217-225	53
VA 51	(CA)7	AAAGCTTTCCATTGTGTGTCGG	CGCGCAAGTTTGAACCAATCTA	261-275	56
VA 52	(CA)10	TGCGCTTAACGTATGTCTGAGT	ATAACAATGAGTCCCACGTGGT	169-223	56
VA 53	(ATT)7	GGCATTAATCTCTCAACTCTGCAC	CATCCATAACAAGCTGGTGAGC	206-245	56
VA 54	(ATG)8	TGGGACATCAATGGGATAAGAGT	CCCTTCCTCTTCAGCCTCATTT	162-186	57
VA 55	(ATG)7	TGGGACAAAACTAGAGGCTGTC	CCAGCTTCCGGTTGTATGTTTG	171-183	57
VA 56	(ATC)9	GTAGCTTGTGTGTCAGATCGGA	CACATCACCCATTGCAAGTGAG	244-259	57
VA 69	(AG)7	GTTCACGAATTGAAGCGGTTGA	GCGTTTGGCTCATCTTCAACAT	110-136	56
VA 70	(AG)10	ATTTCCAGGTTCGAAGCTCCTT	CACTCACATTTTAGTCAGTTGCGA	117-147	55
VA 80	(ACTT)6	GTCCTCCCATGACCACATTCTT	AGTACTCTCTGAGCGGTGTTTG	186-206	57

The total volume of the PCR was 10 μL, including 1 μL genomic DNA (30 ng), 0.5 μL forward M13 primer, 0.5 μL reverse primer, 0.5 μL fluorescent M13 primer, 1 μL 10× buffer, 1 μL dNTP, 0.1 μL Taq enzyme, and 5.4 μL ddH_2_O. The PCR amplification procedure was as follows: 3 min at 94 °C, followed by 30 cycles of 30 s at 94 °C, 30 s at 60 °C, and 30 s at 72 °C, with a final extension at 72°C for 10 min. The PCR products were subsequently detected by an ABI 3730xl Genetic Analyzer Sequencer at Sangon Biotech (Shanghai, China) Co., Ltd., and the outputs were analysed using the software GeneMarker v2.2.0 (SoftGenetics, State College, Pennsylvania, USA).

### Data analysis

The number of alleles (Na), the number of effective alleles (Ne), Shannon’s information index (I), the observed heterozygosity (Ho), the expected heterozygosity (He), and the percentage of polymorphic loci (PPL) were determined to evaluate the genetic diversity of the SSRs and *V. amoena* populations. The genetic differentiation index (Fst) and genetic distance were calculated and principal coordinate analysis (PCoA) and analysis of molecular variance (AMOVA) were performed by GenAlEx 6.5 [28]. A NJ tree was constructed using MEGA X software [29]. Population genetic structure was determined using the model-based program in STRUCTURE 2.3.4 software with a Bayesian approach [30, 31]. The most likely number of populations (K) was identified among 2-24, and 10 interactions were performed for each value of K. The length of burn-in Markov chain Monte Carlo (MCMC) replications was set to 500,000, followed by 100,000 MCMC replications in each run. The optimal K capturing the major structure in the *V. amoena* data was determined using Structure Harvester (http://taylor0.biology.ucla.edu/structureHarvester/) [32, 33]. All tetraploid genotype data were converted into binary data using the POLYSAT v1.2 package in R [34]. Polymorphic information content (PIC) was calculated using the formula PIC = 1-∑*P*_i_^2^, where *P*_i_ is the frequency of the *i-*th allele [35].

## Results

### Characterization of the developed SSR markers

A total of 8799 SSRs with 3 or more di-, tri-, tetra-, penta-, and hexa-nucleotide repeat units were identified in the enriched sequences of the *V. amoena* genome. The sequencing data generated in the present study have been deposited in the National Center for Biotechnology Information (NCBI) Sequence Read Archive (SRA) database (PRJNA742214). Among the SSRs, 2089, 3878, 2055, 533, and 244 were di-, tri-, tetra-, penta-, and hexa-nucleotides SSRs, respectively (Table 3). Of the dinucleotide motifs, AT/TA (25.64%) was the most abundant, followed by AG/TC (20.84%), AC/TG (20.44%), CA/GT (18.60%), and CT/GA (13.58%). The CG/GC motif was the least frequent (0.90%) dinucleotide. Of the trinucleotide motifs, AAC/TTG (14.75%) was the most abundant, followed by CAA/GTT (14.13%), ACA/TGT (13.54%), AAG/TTC (6.13%) and AGA/TCT (6.06%). The remaining trinucleotide motifs were present in less than 5% of the total. Of the tetra-, penta- and hexa-nucleotide motifs, CATA/GTAT (5.89%), ATAAT/TATTA (2.81%), and AAAAAG/TTTTTC (5.73%) were the most abundant, respectively (Table S1). The average length (bp) of di-, tri-, tetra-, penta- and hexa-nucleotide SSRs was 12.67, 13.25, 12.64, 15.87 and 19.30, respectively. The repeat number of 3035 SSR motifs (34.49%) was four, the repeat number of 2601 SSRs (29.56%) was three, and the repeat number of 2026 SSRs (23.03%) was five. The repeat numbers of 551 (6.26%), 199 (2.26%), and 142 (1.61%) SSRs were six, seven, and eight, respectively. The distribution frequency of the other 245 SSRs was less than 1% (Table 4).

**Table 3:** Distribution characteristics of SSR motifs in this study

Repeat type	Number	Percentage (%)	Types of Motif	Average length (bp)
Dinucleotide	2089	23.74	12	12.67
Trinucleotide	3878	44.07	60	13.24
Tetranucleotide	2055	23.35	196	12.64
Pentanucleotide	533	6.06	210	15.87
Hexanucleotide	244	2.77	160	19.30
Total	8799	100.00	638	14.74

**Table 4:** Repetition times and distribution frequency of each SSR repeat unit

Repeat type	Repetition times												
	3	4	5	6	7	8	9	10	11	12	13	14	>15
Dinucleotide	-	-	1266	355	140	120	51	34	19	13	20	9	62
Trinucleotide	-	2861	728	188	57	20	7	8	3	1	-	-	5
Tetranucleotide	1897	115	26	6	1	1	1	-	-	1	1	-	6
Pentanucleotide	481	44	4	1	1	-	-	-	-	-	-	-	2
Hexanucleotide	223	15	2	1	-	1	-	-	-	-	-	-	2
Total	2601	3035	2026	551	199	142	59	42	22	15	21	9	77
Distribution frequency (%)	29.56	34.49	23.03	6.26	2.26	1.61	0.67	0.48	0.25	0.17	0.24	0.10	0.88

For the 21 polymorphic SSR primers, the average allele number (Na) was 3.533, ranging from 3.250 to 15.542 (Table 5). The number of effective alleles (Ne) ranged from 2.680 to 9.751, with an average of 2.521 alleles. Shannon’s information index (I) ranged from 0.999 to 2.655, with an average of 0.930. The average observed heterozygosity (Ho) value was 0.713, ranging from 1.000 to 2.000. The expected heterozygosity (He) values ranged from 0.642 to 1.551, with an average of 0.485. The Fst ranged from 0.029 to 0.140, with an average of 0.384, and the average value of Nm was 2.119, ranging from 1.538 to 8.380. Meanwhile, the PIC ranged from 0.896 to 0.968, with an average of 0.931.

**Table 5:** Genetic diversity index of the used SSR markers in *V. amoena*

Primers ID	Na	Ne	I	Ho	He	F_st_	N_m_	PIC
VA1	3.958±0.363	3.227±0.232	1.223±0.086	1.083±0.058	0.703±0.043	0.066	3.529	0.899
VA4	11.292±1.356	8.328±0.899	2.604±0.247	1.458±0.104	1.161±0.092	0.082	2.811	0.963
VA8	5.500±0.528	4.580±0.491	1.696±0.169	1.375±0.144	0.931±0.094	0.118	1.862	0.905
VA9	15.542±0.815	9.751±0.524	3.454±0.114	1.997±0.003	1.551±0.027	0.056	4.188	0.967
VA11	8.625±0.607	6.070±0.415	2.295±0.140	1.750±0.090	1.200±0.067	0.063	3.711	0.933
VA14	6.333±0.441	5.058±0.346	1.874±0.117	1.542±0.104	1.029±0.065	0.073	3.176	0.914
VA16	4.292±0.467	3.347±0.325	1.286±0.127	1.292±0.095	0.768±0.066	0.050	4.762	0.902
VA19	3.250±0.396	2.680±0.257	0.999±0.103	1.167±0.078	0.642±0.054	0.029	8.380	0.930
VA21	3.375±0.320	3.027±0.265	1.108±0.094	1.042±0.042	0.652±0.042	0.107	2.076	0.903
VA23	5.208±0.519	4.225±0.393	1.476±0.124	1.042±0.042	0.751±0.048	0.140	1.538	0.949
VA25	6.000±0.553	4.841±0.427	1.689±0.129	1.167±0.078	0.854±0.057	0.122	1.807	0.963
VA26	6.375±0.557	4.805±0.408	1.845±0.149	1.583±0.103	1.036±0.076	0.045	5.287	0.912
VA51	5.833±0.462	4.566±0.387	1.674±0.121	1.208±0.085	0.863±0.060	0.099	2.272	0.935
VA52	7.083±0.541	5.470±0.414	1.795±0.099	1.083±0.058	0.838±0.041	0.123	1.782	0.953
VA53	10.625±0.954	6.724±0.473	2.608±0.155	2.000±0.000	1.338±0.039	0.044	5.441	0.942
VA54	9.042±0.768	5.739±0.407	2.299±0.153	1.833±0.078	1.214±0.065	0.051	4.605	0.941
VA55	5.625±0.532	3.822±0.370	1.519±0.139	1.458±0.104	0.883±0.073	0.031	7.777	0.916
VA56	5.625±0.544	3.985±0.354	1.539±0.123	1.333±0.098	0.861±0.062	0.038	6.412	0.924
VA69	9.458±0.983	5.641±0.512	2.242±0.192	1.749±0.092	1.163±0.078	0.040	6.005	0.953
VA70	11.583±1.085	6.982±0.634	2.655±0.202	1.792±0.085	1.277±0.077	0.074	3.115	0.968
VA80	3.750±0.173	2.991±0.095	1.164±0.037	1.000±0.000	0.658±0.011	0.087	2.636	0.896
Mean	3.533±0.095	2.521±0.062	0.930±0.021	0.713±0.014	0.485±0.010	0.384±0.057	2.119±0.364	0.931

*Note*: Na = No. of Alleles; Ne = No. of Effective; I = Shannon's Information Index; Ho = Observed Heterozygosity; He = Expected Heterozygosity; Fst = (Ht - Mean He) / Ht; Nm = [(1 / Fst) - 1] / 4; PIC= Polymorphism information content

### Genetic diversity and structure of *V. amoena* populations

The genetic diversity of the 24 *V. amoena* populations (n=569) was also assessed, revealing high mean per-population estimates of allele and genetic diversity (Na=3.533; Ne=2.521; I=0.930; Ho=0.713; He=0.485; Table 6). The PPL of the 24 *V. amoena* populations ranged from 54.76% to 83.33%, with an average of 71.33%. The genetic diversity was highest in ZX1141 (Na=4.976; Ne=3.191; I=1.159; Ho=0.810; He=0.570) and lowest in QHA (Na=1.857; Ne=1.526; I=0.583; Ho=0.548; He=0.340). The same pattern was found for PPL, which was also higher in ZX1141 (80.95%) than in QHA (54.76%).

**Table 6:** Genetic variability within 24 *V. amoena* populations detected by SSR markers

Populations ID	Na	Ne	I	Ho	He	PPL (%)
M99	3.952±0.438	2.774±0.271	1.402±0.097	0.786±0.064	0.543±0.046	78.57%
NM03	3.357±0.460	2.433±0.289	0.909±0.101	0.738±0.069	0.485±0.047	73.81%
ZX476	3.548±0.426	2.574±0.273	0.954±0.099	0.762±0.067	0.509±0.047	76.19%
ZX541	4.405±0.558	2.949±0.340	1.062±0.108	0.786±0.064	0.537±0.047	78.57%
ZX562	3.952±0.427	2.766±0.281	1.031±0.099	0.786±0.064	0.536±0.046	78.57%
B514	3.310±0.343	2.428±0.226	0.929±0.087	0.785±0.064	0.513±0.044	78.57%
B515	4.405±0.490	3.108±0.310	1.120±0.100	0.833±0.058	0.574±0.043	83.33%
B516	3.905±0.440	2.851±0.296	1.043±0.101	0.786±0.064	0.541±0.046	78.57%
ZX986	4.619±0.529	3.162±0.352	1.132±0.111	0.760±0.066	0.554±0.050	76.19%
ZX987	3.452±0.439	2.641±0.315	0.954±0.101	0.762±0.067	0.507±0.047	76.19%
ZX1141	4.976±0.583	3.191±0.336	1.159±0.109	0.810±0.061	0.570±0.046	80.95%
N50	3.857±0.521	2.672±0.342	0.960±0.112	0.714±0.071	0.490±0.051	71.43%
STG	2.857±0.394	2.172±0.280	0.811±0.102	0.643±0.075	0.436±0.052	64.29%
YDZ	2.333±0.374	1.823±0.278	0.686±0.101	0.571±0.077	0.375±0.052	57.14%
HEB	3.929±0.486	2.579±0.307	0.974±0.110	0.690±0.072	0.488±0.052	69.05%
XLT	3.548±0.486	2.451±0.313	0.910±0.108	0.690±0.072	0.470±0.051	69.05%
QHA	1.857±0.288	1.526±0.230	0.583±0.088	0.548±0.078	0.340±0.049	54.76%
QHB	2.643±0.390	2.073±0.284	0.770±0.104	0.619±0.076	0.416±0.052	61.90%
SJ	2.762±0.399	2.140±0.297	0.783±0.103	0.643±0.075	0.424±0.051	64.29%
YX	3.048±0.423	2.150±0.282	0.814±0.101	0.667±0.074	0.437±0.050	66.67%
YHT	3.119±0.441	2.207±0.287	0.835±0.107	0.643±0.075	0.438±0.052	64.29%
ZQ	4.214±0.516	2.949±0.303	1.073±0.105	0.786±0.064	0.547±0.047	78.57%
MQ	2.929±0.426	2.125±0.304	0.790±0.108	0.619±0.076	0.416±0.053	61.90%
ZD	3.810±0.507	2.747±0.346	0.987±0.115	0.690±0.072	0.494±0.053	69.05%
Mean	3.533±0.095	2.521±0.062	0.930±0.021	0.713±0.014	0.485±0.010	71.33%

Note: Na = No. of Alleles; Ne = No. of Effective Alleles; I = Shannon's Information Index; Ho = Observed Heterozygosity; *He* Expected Heterozygosity; *PPL* Percentage of Polymorphic Loci

In addition, AMOVA was conducted to evaluate variance components among *V. amoena* populations (Table 7). The results revealed significant differences within and among populations (*P*=0.001). A much greater proportion of the variance was observed within populations (88%), than among populations (12%). Population differentiation (Fst=0.048) was significant (*P*=0.001), and the Nm was 4.958.

**Table 7:** Analysis of molecular variance (AMOVA) for *V. amoena* populations

Source of variance	Degrees of freedom	Sum of squares	Mean square	Variance components	Total variance %	
Among populations	23	808.846	35.167	1.132	12%	Fst=0.048 Nm=4.958 *P*=0.001
Within populations	545	4592.969	8.427	8.427	88%	
Total	568	5401.815		9.559	100%	

Fst: genetic differentiation among populaitoins; Nm = [(1 / Fst) - 1] / 4

**Fig. 1:**
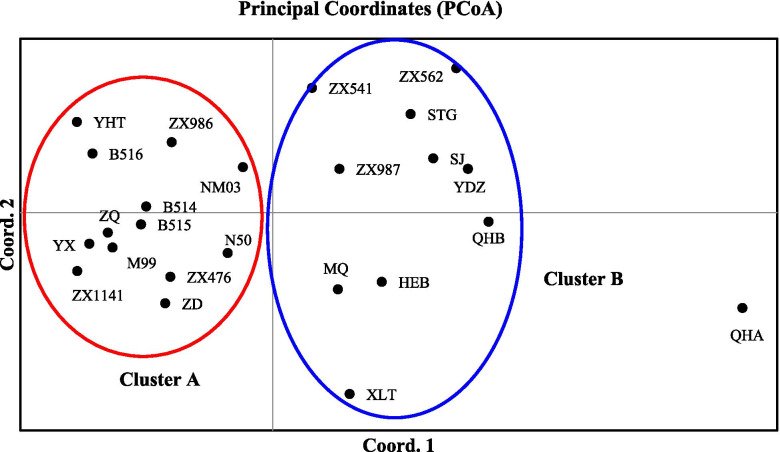
PCoA of the 24 *V. amoena* populations

PCoA, NJ tree construction, and STRUCTURE analysis were conducted to further evaluate the genetic relationships among the 24 *V. amoena* populations. In the PCoA (Fig. 1), the QHA population was separated into a single cluster. The 23 populations were clustered into cluster A and cluster B. Cluster A included the populations from Inner Mongolia (YHT, N50, NM03, ZQ, M99, B514, B515, and B516), Shanxi (YX and ZX476), Heilongjiang (ZD), and Beijing (ZX986 and ZX1141). The other 10 populations were clustered together, including those from Inner Mongolia (STG, YDZ, XLT, and MQ), Heilongjiang (HEB), Shanxi (SJ and ZX562), Beijing (ZX541), Hebei (ZX987), and Qinghai (QHB).

**Fig. 2:**
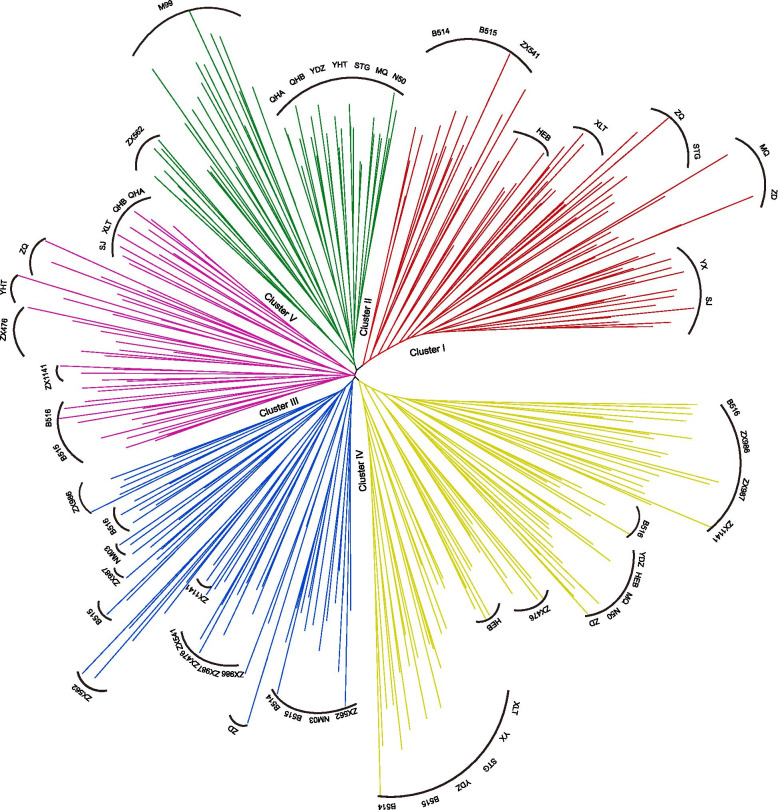
NJ analysis of 24 *V. amoena* populations based on SSR datasets (Cluster I, red; Cluster II, green; Cluster III, blue; Cluster IV, yellow; Cluster V, pink)

Additionally, an NJ tree of *V. amoena* individuals was constructed based on Nei’s genetic distance, and five clusters (Clusters I, II, III, IV, and V) were identified (Fig. 2). Cluster I included the populations from Inner Mongolia (MQ, STG, ZQ, XLT, B514, and B515), Shanxi (YX and SJ), Heilongjiang (HEB), and Beijing (ZX541). Cluster II could be further separated into three sub-clusters: one sub-cluster included the populations from Qinghai (QHA and QHB) and Inner Mongolia (YDZ), the individuals of M99 (Inner Mongolia) formed another sub-cluster, and the individuals of ZX562 (Shanxi) formed the third sub-cluster. ZD (Heilongjiang); ZX987 (Hebei); ZX476 and ZX562 (Shanxi); NM03, B514, B515, and B516 (Inner Mongolia); and ZX541, ZX986, and ZX1141 (Beijing) formed Cluster III. Some individuals from Inner Mongolia (B514, B515, B516, YDZ, MQ, N50, ZD, XLT, and STG), Shanxi (ZX476 and YX), Beijing (ZX986 and ZX1141), Hebei (ZX987), and Heilongjiang (HEB) formed Cluster IV. Cluster V included other individuals from Qinghai (QHA and QHB), Inner Mongolia (B515, B516, XLT, YHT, and ZQ), Shanxi (SJ and ZX476), and Beijing (ZX1141).

**Fig. 3:**
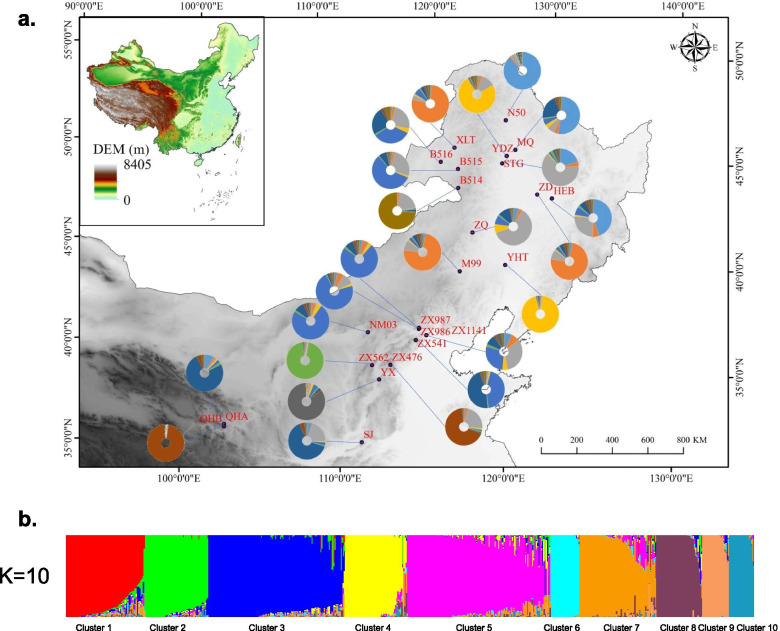
STRUCTURE analysis of the entire SSR dataset (24 populations, n=569). **a** The geographic distribution of the 24 *V. amoena* populations inferred with K=10. The different colours of the pie charts represent the proportions of the populations in the 10 clusters (Table S2). **b** STRUCTURE output with K=10 (Fig. S1) showing the population structure among 569 individuals; vertical lines represent individuals.

For the entire SSR dataset (24 populations, n=569), STRUCTURE analysis yielded the highest likelihood when samples were clustered into 10 groups (K=10, Fig. S1). The populations from Heilongjiang were assigned to cluster 1 (HEB, 44.9%) and cluster 2 (ZD, 74.1%). The populations from Inner Mongolia were assigned to 7 clusters, and the individuals of B514 (73.3%) formed a separate cluster (cluster 10). Only populations YDZ (75%) and YHT (94.1%) were assigned to cluster 4. The other populations were clustered with populations from other regions: N50 (87.5%) and MQ (53%) were assigned to cluster 1; XLT (77.4%) was assigned to cluster 2; M99 (44%), STG (63.5%), and ZQ (61.4%) were assigned to cluster 3; NM03 (73.8%) and B515 (57.5%) were assigned to cluster 5; and ZX541 (45.5%) was clustered into cluster 7. The Beijing populations were all assigned to cluster 5, which included ZX541 (44%), ZX986 (72.1%), and ZX1141 (27.8%). The individuals of ZX1141 (32.7%) were assigned to cluster 3. The individuals of Hebei (ZX987, 73.5%) were assigned to cluster 5 with the Beijing populations. Almost all the individuals of ZX562 (93.9%) formed a separate cluster (cluster 6), and the individuals of YX (84.1%) formed a separate cluster (cluster 9). The other two populations from Shanxi were assigned to cluster 7 (SJ, 63.8%) and cluster 8 (ZX476, 67.8%). The populations from Qinghai were mainly assigned to cluster 7 (QHB, 75.6%) and cluster 8 (QHA 96.1%) (Fig. 3, Table S2).

## Discussion

### The development and polymorphism of SSR markers

Genetic research on *V. amoena* has developed slowly due to a lack of sufficient genetic information and effective molecular marker systems. SSRs are one of the most important marker systems for plant genetic studies with genetic diversity evaluation, marker-assisted selection (MAS) breeding, quantitative trait locus (QTL) mapping, and variety identification and are extensively distributed throughout eukaryotic genomes [36, 37]. However, traditional SSR development methods are labour intensive [13]. At present, SSR markers developed by high-throughput sequencing are reliable and effective [19, 38–41]. Genomic SSRs have not been developed thus far in *V. amoena*, and a new set of highly polymorphic SSR markers was successfully developed in the present study. A total of 8799 SSRs were developed in *V. amoena* at the genome-wide scale, which was far greater than the 1071 EST-SSRs developed by transcriptome sequencing in *V. sativa* [15]. Our work provides a powerful tool for genetic research on *V. amoena* in future breeding programmes and resource conservation. Among the SSR markers, trinucleotide repeats were the most abundant (44.07%) type, similar to the relative proportions of EST-SSR motif types observed in *V. sativa* [15] and *Medicago sativa* [16]. The results indicated that the trinucleotide SSRs in the *V. amoena* genome are mainly located in exon regions. The frequent distribution of trinucleotide repeats in coding regions indicates the effects of selection and evolution [41].

The 21 SSR markers used in this study offered an informative and applicable approach for the evaluation of genetic relationships among the *V. amoena* populations. The genetic diversity parameter values indicated the high polymorphism of the 21 SSR markers. The observed heterozygosity (Ho) and expected heterozygosity (He) values also revealed a high degree of genetic variability among the *V. amoena* populations [11]. The values of PIC, Ho and He were all higher than those of the EST-SSRs reported in *V. sativa* [15]. This could be related to the different methods of SSR marker development and the different genetic backgrounds of various plant species.

### Genetic differentiation and genetic structure of *V. amoena* populations

In the present study, a high level of genetic diversity (I=0.930) was detected among the *V. amoena* populations by the newly developed SSR markers. This genetic diversity was more evident than that detected by SRAP and ISSR markers in a previous report (I=0.397) [2]. Two reasons for this difference are that SSR markers are more effective than the other two types of markers [42] and more natural populations were examined in the present study. Among the populations, those from Qinghai Province showed a lower level of genetic diversity, which may be due to their unique geographical location on the Qinghai-Tibet Plateau. The populations from tall mountain areas with high forest coverage at approximately 40°N had a higher level of genetic diversity. Genetic variation within the populations (88%) was higher than that among the populations (12%) in this study. The results were consistent with the characteristics of outcrossing species [43, 44], which can be attributed to allogamous reproductive behaviour. The variation in *V. amoena* mainly comes from intrapopulation variation, confirming that *V. amoena* is a cross-pollinating plant.

The 24 *V. amoena* populations could be separated into three clusters via PCoA. The populations were mainly separated by habitat, i.e., mountain meadow, *Leymus chinensis* steppe, and undergrowth on mountains. The results indicated that the elevation of the geographical origin may be an important factor explaining the clustered pattern of *V. amoena* and that special habitat is another important factor. Similar results were found in the STRUCTURE analysis. The inferred subpopulations were broadly separated based on the best K value (K=10). The populations were mainly clustered among *Leymus chinensis* steppe, mountain areas with high forest coverage, and the Qinghai-Tibet Plateau. The results showed that the clusters of *V. amoena* were impacted by different landforms and the special topography of the Qinghai-Tibet Plateau. It would be worth exploring how the special topography affects the genetic differentiation of *V. amoena* in the future.

Additionally, the NJ analysis of *V. amoena* based on the entire SSR dataset revealed five major groups and showed an interesting pattern. The individuals from the populations on mountains were clustered with the populations from the Qinghai-Tibet Plateau. The other populations from the mountains and *Leymus chinensis* steppe were gathered in three clusters. The clustered pattern in the NJ analysis did not show clear boundaries among the different habitats and elevations. The high gene flow (Nm= 4.958) also weakened the differentiation among the *V. amoena* populations. The results indicated that the genetic structure of *V. amoena* populations was complex and affected by many factors, which needs further analysis. This might be due to the special climatic conditions, habitats, and geomorphic conditions [2].

In conclusion, our results confirmed that the *V. amoena* populations in China contained a high level of genetic diversity. There is a tendency for the genetic structure of the populations to be correlated with geographical origin and comprehensive environmental factors. Our findings and the SSRs newly developed in the present study provide a strong tool for breeding improvement and germplasm resource conservation in *V. amoena*.

## Supplementary Information

**Additional file 1: Table S1.** The repeats number of different SSR motifs. **Table S2.** The proportion of each population in the genetic structure analysis. **Figure S1.** The best K-value of the genetic structure based on STRUCTURE analysis.

## Data Availability

The data supporting this article are included within the article and its additional files. The original sequencing data generated in the study have been deposited into the National Center for Biotechnology Information (NCBI) Sequence Read Archive (SRA) database (https://www.ncbi.nlm.nih.gov/bioproject/PRJNA742214).

## Abbreviations

SSR: Simple sequence repeat
PIC: Polymorphism information content
Na: Number of alleles
Ne: Number of effective alleles
I: Shannon’s information index
Ho: Observed heterozygosity
He: Expected heterozygosity
PPL: Percentage of polymorphic loci
AMOVA: Analysis of molecular variance
UPGMA: Unweighted pair-group method with arithmetic means
