# Supplementary figures and images for: Genetic diversity and population structure analysis in a large collection of Vicia amoena in China with newly developed SSR markers
Source: BMC Plant Biol. 2021 Nov 20;21:544. doi: 10.1186/s12870-021-03330-w (PMC8605504; doi:10.1186/s12870-021-03330-w)

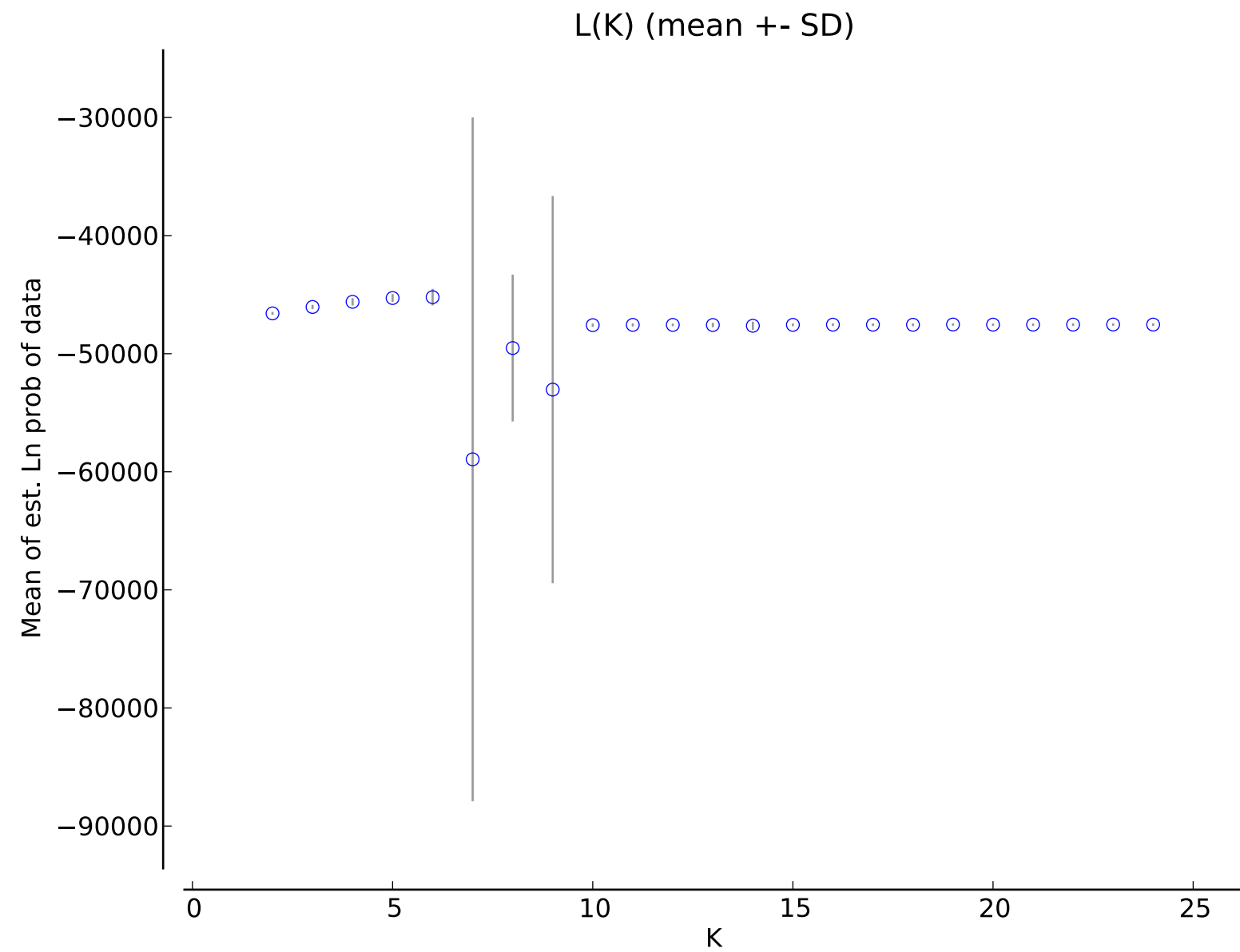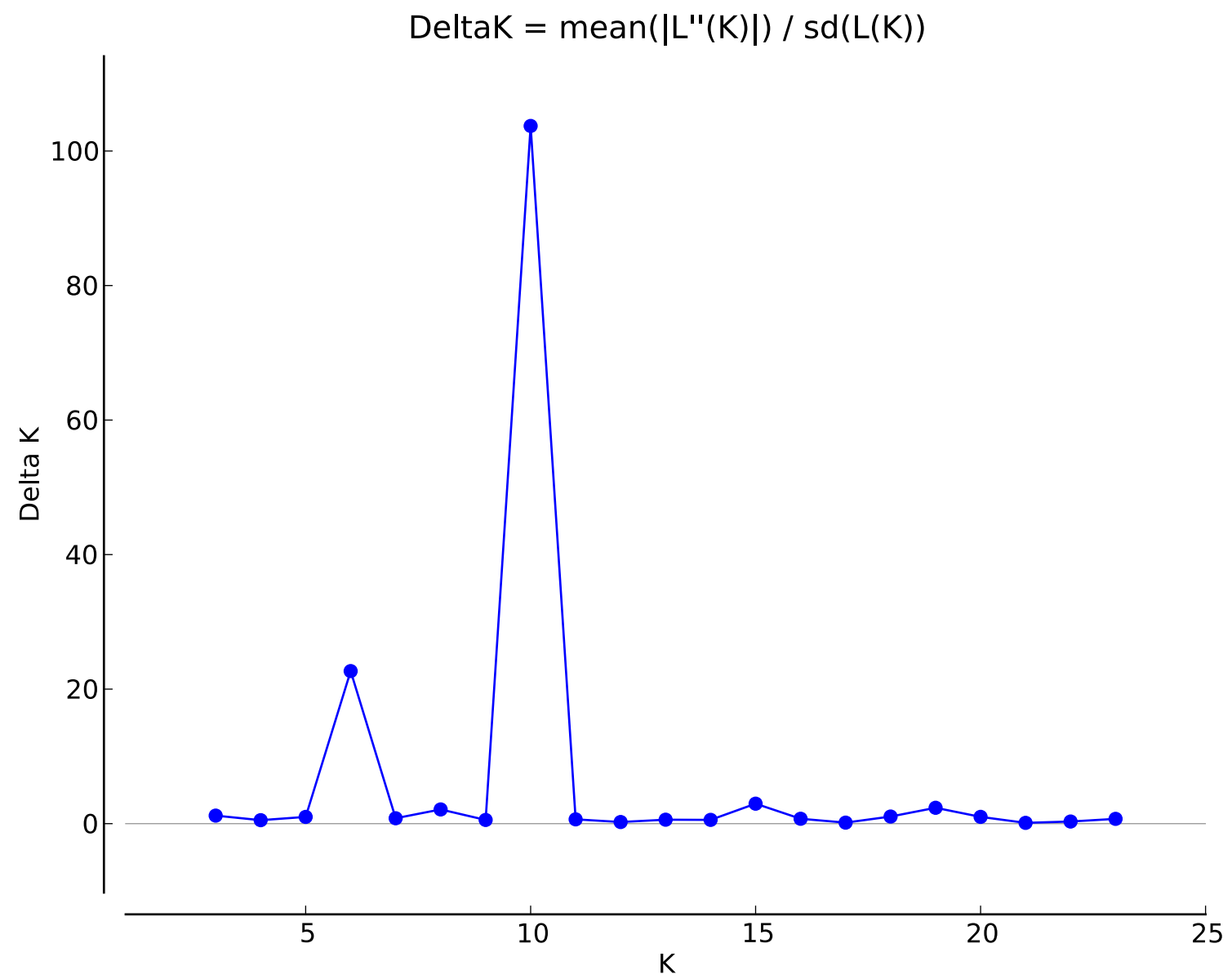

Supplement: Supplementary file 1 — Additional file 1: Table S1. The repeats number of different SSR motifs. Table S2. The proportion of each population in the genetic structure analysis. Figure S1. The best K-value of the genetic structure based on STRUCTURE analysis. [file 12870_2021_3330_MOESM1_ESM.zip › 12 Fig. S1.pdf]
